# Editorial: Cardiometabolic diseases in understudied populations

**DOI:** 10.3389/fepid.2023.1160108

**Published:** 2023-05-11

**Authors:** Liliana Aguayo, Andrea López-Cepero, Xiao Tan, Mayra L. Estrella, Victor W. Zhong

**Affiliations:** 1 Hubert Department of Global Health, Rollins School of Public Health, Emory University, Atlanta, GA, United States,; 2 Department of Epidemiology, Rollins School of Public Health, Emory University, Atlanta, GA, United States,; 3 Department of Big Data in Health Science, Zhejiang University School of Public Health and Department of Psychiatry, Sir Run Run Shaw Hospital, Zhejiang University School of Medicine, Hangzhou, China,; 4 Department of Medical Sciences, Uppsala University, Uppsala, Sweden,; 5 Department of Epidemiology, Human Genetics, and Environmental Sciences, School of Public Health, University of Texas Health Science Center at Houston, Brownsville, TX, United States,; 6 Department of Epidemiology and Biostatistics, School of Public Health, Shanghai Jiao Tong University School of Medicine, Shanghai, China

**Keywords:** cardiometabolic, underrepresented and minority groups, cardiovascular disease, atheroclerotic cardiovascular disease, global cardiometabolic risk

## Editorial on the Research Topic

### Cardiometabolic diseases in understudied populations

Cardiovascular diseases (CVD) remain the leading cause of death worldwide. Every year, about 17.8 million people die from CVD, accounting for nearly 32% of all deaths (1). Notably, the burden of CVD morbidity and mortality is not equally distributed. Low and middle-income populations, and certain historically underrepresented and underserved populations living in high-income countries, bear a disproportionately greater and growing burden of CVD (2).

However, high-quality scientific studies of structural, clinical, and lifestyle determinants of CVD among the most vulnerable populations are scarce. The fundamental knowledge that informs CVD prevention, diagnosis, and treatment is mainly derived from epidemiological data collected in high-income countries amongst high-income white, middle-aged, and older adults living in predominantly urban settings (2). The limited diversity in CVD research reveals a critical gap in our knowledge base that introduces important challenges for the development of clinical guidelines, interventions, and policies that aim to reduce the global burden of CVD and, ultimately, address health disparities.

In this Research Topic, we sought to shed light on this important knowledge gap and contribute to increasing the representation of underserved populations in CVD research. We bring together four studies that present the CVD experiences of four historically underrepresented and underserved populations: Black and Asian Americans living in the U.S., and adults at risk for developing or already with CVD living in Nepal and Haiti.

In the U.S., multiple profound overlapping social inequalities affect CVD mortality. In this context, Bell et al. examined whether the associations between self-employment status and hypertension varied by race, sex, and level of education. Using data from the National Health and Nutrition Examination Survey (NHANES), a nationally representative dataset of non-institutionalized U.S. adults, Bell et al. raised awareness of the hypertension risk that was associated with self-employment status, and how this association varied across different levels of educational attainment among US Black adults. Their findings help us to better understand the intersectionality between race, educational attainment, and types of employment, and their contribution to the disproportionally higher hypertension rates in this underrepresented population.

Similarly, using data from NHANES, Thomas and Leak described the association of the Healthy Eating Index-2015 score (a measurement used to quantify adherence to the Dietary Guidelines for Americans) with obesity risk among Asian Americans. Their study highlights cross-cultural differences in otherwise considered “traditional dietary risk factors” (Thomas and Leak). Their unexpected findings—specifically those that did not align with previous examinations of whole grains, sodium and obesity risks among U.S. adults (3)—remind us that one size does not fit all. This study highlighted the urgent need for culturally relevant assessments of overall dietary quality (and its components) to inform clinical practice, policies, and interventions geared toward Asian American adults.

Beyond the U.S. populations, studies by Peoples et al. and Yan et al. provide two examples of the barriers that individuals with CVD in Nepal and Haiti, respectively, are forced to face and the consequences of receiving sub-optimal healthcare services (e.g., health care centers with a limited number of health care professionals, low medication supplies, and poor diagnostic capacity). Peoples et al. used quantitative and qualitative data to document the dissatisfaction with primary healthcare services among adults (aged ~55 years) with heart disease, hypertension, stroke, and/or diabetes. Although Nepalese policies regulate free access to basic healthcare services in primary healthcare centers (Thomas and Leak), this policy is not always translated into practice. Peoples et al. reported that most study participants with CVD perceived the cost of medications and treatments as “expensive or very expensive”. Qualitative complementary analyses revealed that patients with CVD are aware of serious gaps between policy and practice. Patients with CVD perceived that primary healthcare centers often lack medicines and equipment needed for CVD diagnosis and management, are regularly understaffed, and the available staff tends to have a poor rapport with their patients (Peoples et al.).

In contrast, Yan et al. discussed evidence of the benefits that may be achieved by providing access to polypills for atherosclerotic CVD (ASCVD) to adults (aged 40 years and older) in Haiti. Using cross-sectional data from adults who participated in the Haiti CVD Cohort, the authors estimated the number of adults who would be eligible for ASCVD polypills. This study quantified the potential ASCVD events that could be avoided if polypills were implemented for primary and secondary ASCVD prevention over 5 years (Yan et al.). The authors found that incorporating a nationwide polypill strategy could prevent about 32% of ASCVD events in Haiti (Yan et al.). Taken together, these studies by Yan et al. and Peoples et al. documented the potential CVD events that could be prevented by enhancing access to adequately equipped healthcare centers with qualified medical care professionals, diagnostic equipment, and medical supplies.

In conclusion, this Research Topic brings together four studies in which investigators implemented a variety of methodologies to examine structural, clinical, and lifestyle determinants of CVD outcomes in the U.S., Nepal, and Haiti. These studies highlight the importance of considering cultural, environmental, and socioeconomic differences, and devoting more resources to learning about the unique assets and challenges faced by underserved and underrepresented populations. Increasing representation and diversity in CVD research will enable us to develop more informed, tailored responses that meet the needs of underrepresented communities in all countries, build upon their strengths, and ultimately facilitate the pursuit of healthy lives for all. We hope this Research Topic will motivate investigators, clinicians, policymakers, funding institutions, and organizations to correct this clinical and epidemiological CVD research deficiency.
